# Higher vegetable intake and vegetable variety is associated with a better self-reported health-related quality of life (HR-QoL) in a cross-sectional survey of rural northern Ghanaian women in fertile age

**DOI:** 10.1186/s12889-018-5845-3

**Published:** 2018-07-27

**Authors:** Fusta Azupogo, Judith A. Seidu, Yakubu Balma Issaka

**Affiliations:** 1 Department of Family and Consumer Sciences, Faculty of Agriculture, University for Development Studies, P. O. Box TL 1882, Nyankpala Campus, Tamale, Ghana; 2 Department of Agri-business Management and Finance, Faculty of Agribusiness and Communication Sciences, University for Development Studies, P. O. Box TL 2352, Nyankpala Campus, Tamale, Ghana

**Keywords:** Vegetable intake, Vegetable variety, Physical health, Mental health, Quality of life, Women in fertile age

## Abstract

**Background:**

A higher vegetable intake plays an important role in promoting general health and well-being, but there is a dearth of data on the independent effect of vegetable intake on health-related quality of life (HR-QoL). This study contributes to evidence on the independent effect of vegetable consumption on HR-QoL among women in fertile age.

**Methods:**

A cross-sectional study of a sample of rural women in fertile age (15–49 years, *n* = 187), randomly selected from 6 rural communities in the Tolon and Savelugu Districts, Northern Region of Ghana. Vegetable consumption in the past month was assessed with a 27-item semi-quantitative food frequency questionnaire; self-reported HR-QoL with the Short Form Health Survey (SF-36); nutritional status with anthropometry; household food security with the household hunger scale (HHS) and demographic and socio-economic related covariates with a pre-tested semi-structured questionnaire using face-face interviews. Generalised Linear Models were fitted to assess adjusted mean scores and their 95% confidence intervals (95% CIs) by terciles of vegetable intake and vegetable variety score (VVS) for the HR-QoL, its physical health (PH) and mental health (MH) domains and the SF-36 subscales.

**Results:**

The mean vegetable intake of the women was 324.6 ± 196.1 g/day. The mean scores of the HR-QoL, PH and MH were 69.5 ± 13.6, 72.6 ± 17.4 and 66.4 ± 12.6 respectively. The alpha Cronbach measure of reliability for the HR-QoL, PH and MH were 0.78, 0.75 and 0.62 respectively. After adjusting for potential confounders such as age, body-mass-index (BMI), parity, educational status, occupation, marital status, HHS and household asset index, we observed an increasing trend across terciles of vegetable intake in the past month for the HR-QoL (*P*-trend = 0.0003), PH (*P*-trend = 0.02), MH (*P*-trend = 0.001) as well as the physical functioning (*P*-trend = 0.01), role-physical (*P*-trend <.0001), and role emotional (*P*-trend <.0001) domains of the SF-36. The multivariate model of the results also showed a significant increasing trend in the adjusted mean scores of the HR-QoL (*P*-trend = 0.04), MH (*P*-trend = 0.001) as well as 4 subscales of the SF-36 [role-physical (*P*-trend = 0.02), role-emotional (*P*-trend = 0.05), emotional well-being (*P*-trend = 0.002) and vitality (*P*-trend <.0001)] across terciles of the VVS.

**Conclusion:**

The results of the present study suggest a potential beneficial role of high vegetable intake and consumption of more varied vegetables on HR-QoL. Further research is needed to determine the mechanisms driving these influences.

**Electronic supplementary material:**

The online version of this article (10.1186/s12889-018-5845-3) contains supplementary material, which is available to authorized users.

## Background

The World Cancer Research Fund/American Institute for Cancer Research recommends consumption of at least 400 g/day (five portions/servings) of a variety of non-starchy vegetables and fruits every day for the prevention of cancer [1]. It is estimated that consumption of vegetables in Ghana is about 50.1 kg/person/year (approximately 137.3 g/person/day) [2]; this is relatively low compared to the recommended minimum daily requirements of 400 g/day for cancer prevention [1]. According to the 2014 Ghana demographic health survey, the average rate of consumption of fruits and vegetables by women in fertile age (15–49 years old) is 3 days out of the previous 7 days [3]. However, analysis by Ruel et al. [2] shows that vegetable consumption in Ghana is more than twice the consumption of fruits per kilogram/person/year (50.1 vs 23.5 kg/person/year).

According to the Ghana Statistical Service [4], less than 3% of fruit consumption in Ghana is home-grown with only 1.3% of fruit consumption in the northern savannah zone being home-grown. Overall, fruit consumption in northern Ghana is poor [5] as fewer fruits are cultivated in the north compared to the south [4]. In the Northern Region of Ghana, Florkowski et al. [6] showed that the consumption of home-grown fruits was only 0.1% while the share of home-grown vegetable consumption was 35.1%. Even in the urban city of Tamale in Northern Ghana, consumers were more likely to spend on vegetables but not on fruits [7]. Although the preference for vegetables is partly attributed to the high cost of fruits in the region [6, 7], it may also be related to the cultural dietary patterns of consuming starchy staples with vegetable soups and stews [8].

Vegetables are commonly cultivated in northern Ghana [9], but mostly for home use mainly in the rural settings [4, 10]. Northern Ghana accounted for about 30% of all vegetables harvested in Ghana between October 2012–October 2013 [4]. Although most vegetables are cultivated in rain-fed cultivation systems, they are also commonly cultivated through irrigation during the dry season in many rural communities [9]; thereby ensuring year-round access to fresh vegetables in these rural communities.

Most existing studies have examined the combined effect of fruit and vegetable consumption on self-reported health-related quality of life (HR-QoL) with mixed results. For instance, while Södergren et al. [11] found a significant positive association between fruit and vegetable intake and self-rated health among older Australian adults, Kwon et al. [12] found a significant association between fruits and vegetable intake and the HR-QoL among older Chinese but not older Hispanics nor blacks in New York. Also, Chai et al. [13] in a Hawaii adult population found no significant association between daily fruit and vegetable intake and HR-QoL; suggesting a lack of consistency in the relationship between vegetable/fruit intake and the HR-QoL.

Nonetheless, Gong et al. [14] found that breast cancer survivors consuming a minimum of 250 g/day of vegetables had significantly higher scores for HR-QoL as well as many of the sub-scales of the HR-QoL compared to their peers who consumed less. Matthews et al. [15] also found vegetable intake to be associated with the general health subscale of the HR-QoL among older Australian adult men and women.

The self-reported HR-QoL is a conceptualisation reflecting an individual’s physical and mental well-being and has emerged as an essential consideration in the prevention and treatment of diseases. The elderly population or populations with chronic diseases such as cardiovascular diseases, arthritis, pulmonary diseases, and cancer have been the primary focus of research on HR-QoL [11–13, 16]. However, its repeatability in other population groups remains to be explored.

Considering the lack of data on the independent effect of vegetable intake on the HR-QoL, we conducted a cross-sectional study of vegetable consumption among rural Northern Ghanaian women (15–49 years) in fertile age with the primary aim of assessing the association between vegetable intake in the past month and self-reported HR-QoL and its subscales. Additionally, we analysed the association between vegetable variety and the self-reported HR-QoL among the rural women in fertile age. Such data may be relevant in future community and population-based programs or policies for improved mental and physical health-related quality of life.

## Methods

### Study design

A cross-sectional study design was used; in this design, exposure (vegetable consumption and variety) and outcome variable (self-reported quality of life) were measured simultaneously. The method of data collection involved face-to-face interviews. All tools for data collection were pre-tested in a pilot study (in a non-survey community) conducted in March 2016; the main survey was conducted between April–May 2016.

### Ethical consideration

Permission was obtained from the opinion leaders of each community included in the survey. Thumb-printed informed consent was obtained from each of the women, and the husband and/or the household head witnessed the informed consent by thumb-print before interviews. Participation in the survey was entirely voluntary, and participants had the option of declining to answer specific questions or to leave the entire questionnaire blank if they did not wish to participate; however, none opted out of the interviews. The study protocol was approved by the Scientific Review Committee of the School of Allied Health Sciences, University for Development Studies, Ghana. The completed questionnaire did not contain any identifying information about the individual subjects. All data were kept confidential, and data protection was observed at all stages of the study.

### Study area

The study was carried out in the Tolon and Salvelugu Districts in the Northern Region of Ghana. Both districts share a boundary with the Kumbugu District and are about 30 Km each away from Tamale, the regional capital. The districts have a unimodal rainy season between May and October, and the vegetation is guinea savannah with a dry season which starts in November and ends in April with maximum temperatures occurring towards the end of the dry season [17]. Most of the inhabitants are subsistence farmers and the chief crops cultivated include maize, yam, groundnut, cowpea and rice [18]. Common vegetables grown in the districts include amaranth (*Amaranthus spp*.), okra (*Abelmoschus esculentus*), jute mallow (*Corchorus olitorius*), Kenaf/Roselle (*Hibiscus sabdariffa*), tomato (*Solanum lycopersicum*), pepper (*Capsicum Spp.*), and garden egg (*Solanum melongena*). Although the vegetables are grown in rain-fed agriculture, households in the sampled communities also cultivate vegetables in the dry season (November to May) under irrigation.

### Subjects

The study population consisted of women in fertile age (15–49 years of age) from 6 rural communities in the Tolon and Savelugu districts in the Northern Region of Ghana. The main inclusion criteria were: apparently healthy woman aged 15–49 years, not currently lactating and not pregnant (self-reported).

### Sample size and sample selection

Six (6) survey communities were randomly selected from a list of 15 communities with access to irrigation and therefore, year-round access to fresh vegetables. These communities had been sensitised on the need to consume more fresh vegetables earlier. A sample size of 186 respondents was estimated based on the one random sample formula [19] considering a one-sided hypothesis, a power of 80%, Type I error of 5%, HR-QoL standard deviation of 0.55 from a previous study [20] and a change in HR-QoL of 10%. Sampling units were households, and the sample size from each community was selected proportional to the total number of households in the community and ranged from 26 to 43. Households were selected systematically with every second household, with the unit committee chairman’s house as a starting point, included in the sample. Upon entering a household, one adult female, who met the inclusion criteria, was selected through a lottery; in a few cases, respondents were purposively selected where there was only one qualified adult female in the household (*n* = 37).

### Exposure assessment

Vegetable intake in the past month was assessed through a pre-tested 27-item (Table 1) semi-quantitative food-frequency questionnaire (FFQ). The FFQ was explicitly developed to cover vegetables commonly available and consumed in the study area. Special care was also taken to probe for information on additional vegetables eaten during the one-month reference period which were not captured by the FFQ, but we found none. In the FFQ, respondents were asked to recall their frequency of intake in the past 30 days for each listed vegetable. Frequency variables included the following: never, once a month, 2–3 times a month, once a week, 2–3 times a week, 4–6 times a week and every day. Each food item in the FFQ included a typical portion size and portion sizes were estimated with standard household measures like ladles, plates, bowls and spoons, food models and picture aids. Vegetable consumption in the past month was determined by multiplying the estimated portion size by the consumption frequency. Respondents were subsequently ranked into terciles (low, moderate and high) of vegetable intake in the past month.

**Table 1:** List of vegetables included in the food frequency questionnaire

Name of vegetable	
Alefu (Amaranth)	
Fresh Kenaf/roselle	
Dry kenaf/roselle	
Dry okra fruit	
Fresh Okra fruit	
Okra leaves	
Cassava leaves	
Cowpea leaves	
Jute mallow	
Bitter leaves	
Dried baobab leaves	
Dried baobab leaves	
Pumpkin fruit	
Pumpkin leaves	
Garden eggs	
Africa might shade	
Tomatoes	
Moringa leaves	
Fresh red pepper	
Dried red pepper	
Onions	
Carrot	
Cabbage	
Sweet pepper	
Sprout onion	
Cucumber	
Unripe beans	

Lastly, a vegetable variety score (VVS) similar to the food variety score [21] comprising the different varieties of vegetables consumed within the 1-month reference period was constructed. In the VVS, a score of 1 was assigned for each variety of vegetable consumed; consumption of the fresh and/or dried forms (e.g. fresh okra fruit and/or dried okra fruit), fruits and/or leaves (e.g. okra fruit and/or okra leaves) of a vegetable were each assigned a score of 1. The VVS ranged from a minimum of 0 to a maximum of 20 and did not consider a minimum intake (in gram).

### SF-36v2 and quality of life

Participants’ self-reported physical and mental health-related quality of life (HR-QoL) was assessed with the Short Form Health Survey (SF-36v2) [22–24]. The SF-36v2 is a 36-item questionnaire, widely validated and popularly used in determining the subjective quality of life of patients and the general public. The SF-36v2 contains 36 items which measure eight multi-item parameters of health status: physical functioning, role limitations due to physical health problems (role-physical), bodily pain, general health perceptions, vitality/energy, social functioning, role limitations because of emotional problems (role-emotional) and emotional well-being. The summated scores for the first four domains reflect physical health (PH) and the latter 4 reflect mental health (MH) [23]. The summated score for all the eight subscales represents the HR-QoL. Scores for the eight scales were calculated with the QualityMetric Health Outcomes™ Scoring Software 5.0 and transformed to a scale from 0 (the worst possible condition) to 100 (the best possible condition). For example, a physical functioning score of 100 means a complete ability to perform any physical function while a score of 0 means complete inability to perform any physical function. The questionnaire was administered through a face-face field interview with trained research assistants.

### Anthropometry

Weight and height of the women were measured according to standard procedures [25]. Height was measured to the nearest 0.1 cm with a microtoise (Bodymeter 208; Seca) while weight was measured to the nearest 0.1 kg with an electronic scale (UNIscale; Seca). A known weight was used to calibrate the scale on each measurement day. The average of duplicate measurements was used to compute body-mass-index as weight (kg)/height (m^2^). Subjects were subsequently classified as underweight, normal weight, overweight and obese with the recommended cut-off points [26].

### Household hunger scale (HHS)

The Household Hunger Scale (HHS) developed by FANTA [27] was used to access the level of respondents’ household food security. Following standard coding, each of the three items in the household hunger scale (HHS) were coded 0, 1 or 2 corresponding to hunger frequencies of “never”, “rarely or sometimes” and “often” respectively. This resulted in total scores ranging from 0 to 6 based on which households were categorised into three standard groups: little/no household hunger (HHS ≤ 1); moderate household hunger (HHS 2–3); severe household hunger (HHS 4–6) [27].

### Wealth index

We constructed terciles of a household assets index using household durables assets [28]. Household durables included in the assets index were radio, television, refrigerator, electric iron, box iron, computer (desktop or laptop), mobile phone, sewing machine, generator, mattress, bicycle, motorcycle, car, open truck/tractor, canoe, jewellery/ornaments and gas stove. The index reflected household wealth in relation to household ownership of durables. The Kaiser-Meyer-Olkin (KMO) measure of sampling adequacy verified the use of Principal Component Analysis for the analysis; KMO = 0.81 (good according to Field [29]) and all KMO values for individual variables were > 0.50 which is the acceptable limit [29].

### Other covariates

Demographic and socio-economic related covariates were assessed with a pre-tested semi-structured questionnaire. The demographic and socio-economic covariates included household dependency ratio which was computed as the sum of number of children ≤10 years and elderly ≥60 years divided by total household size [Σ sum (≤ 10 years children + ≥ 60 years elderly)/(Total household size)]; religion, ethnicity, marital status, educational status of sampled woman and household head, occupation of woman and household head, parity of sampled woman, history of any chronic disease or condition (hypertension, diabetes, cancer), smoking and drinking status. Although a 3-point scale was included to classify each woman as never, occasional, and regular drinkers (≥ 5 serves of drink per week), none of the women drunk alcohol. Furthermore, smoking status was captured as never smoked, ever smoked and current smoker but was not found to be an influential covariate in the present study. A voter’s identification card or national health insurance card was used to verify the ages of the women and their household heads.

### Statistical analysis

#### Recoding of variables for statistical analysis

Occupation was re-categorised as farmer and other (petty trader, formal paid job and informal paid job) as most of the women, as well as their household heads, were farmers. Similarly, the educational status of the women and their household heads was recoded as literate (ever-schooled) and non-literate (never-schooled) as most of them had never been to school. Although gram of vegetable intake in the past month is a continuous variable, terciles of the gram of vegetable intake in the past month were preferred for the statistical analysis as recall bias is the main limitation of an FFQ. Our choice of terciles instead of quartiles or quintiles was based on the rule of thumb [30, 31] to have a minimum sample of 50 subjects in each category when categorizing continuous exposure variables. Although categorization of data may lead to loss of statistical power [32]; the approach is commonly used in epidemiological studies [30, 33] to make public health interpretations that can better be understood by the general population.

### Analysis

Means (standard deviation-SD) and percentages were calculated for continuous and categorical data respectively. One-way Analysis of Variance (ANOVA) was used to determine the trend across terciles of vegetable intake for continuous data while the association between categorical variables and terciles of the vegetable intake was appropriately analysed with Chi-square and Fisher Exact Test.

Generalised Linear Models were fitted to assess adjusted mean scores and their 95% confidence intervals (95% CIs) for the HR-QoL, PH and MH components as well as the SF-36 subscales scores according to terciles of vegetable intake in the past month. Hierarchical multivariate models adjusted for potential confounders which were a priori selected based on literature and included age, educational status, marital status, BMI, occupation, parity, household food security, and history of chronic diseases [11–13, 34, 35]. In the multivariate model, we adjusted for age of woman, body-mass-index (BMI), parity, educational status of participant (literate/non-literate), occupation of participant (farmer/other), marital status of participant (never married, currently married and formerly married), age of household head, household hunger scale and terciles of household asset. A *post-hoc* procedure (Tukey-Kramer adjustment) was used to ascertain if the mean scores were significantly different by pairs. Tests of linear trend (*P*-trend) across successive terciles were conducted by assigning the median scores to each tercile and treating this consumption as a continuous variable. Potential effect modification by age and BMI was assessed by including an interaction term in the regression models and significance ascertained at 5% level; however, none were found to be statistically significant. The analysis was repeated with terciles of the VVS. All statistical analyses were done with SAS 9.3 (SAS Institute Inc., Cary NC.) and a two-tailed *P-*value ≤0.05 at 95% confidence interval was considered statistically significant.

## Results

### Population characteristics

Table 2 shows the characteristics of the study population according to terciles of the HR-QoL. The study population had a mean age of 38.1 ± 11.2 years and a mean BMI of 22.2 ± 9.0 kg/m^2^. Participants had a mean vegetable intake of 324.6 ± 196.1 g/day and a mean VVS of 10.5 ± 3.1. The mean frequency of vegetable intake in the past month by participants was 8.3 ± 2.6. None of the participants drank alcoholic beverages and only 1.6% ever smoked. Most participants (87.2%) as well as their household heads (85.6%) had never been to school (non-literate) and were generally farmers. A majority (91.4%) of participants were currently married and lived with their spouses. The most prevalent religion among participants was Islam which accounted for 93.0% of participants. The mean number of births (parity) of participants was 4.6 ± 2.2, and the mean dependency ratio was 0.4 ± 0.2. The prevalence of hypertension and diabetes were 5.4 and 1.1% respectively and did not vary across terciles of vegetable intake. The prevalence of moderate and severe household hunger in the last month was 17.1 and 3.7% respectively; the prevalence did not differ between terciles of vegetable intake. Participants in the uttermost tercile of vegetable intake were more likely to have a higher frequency of vegetable intake in the last month, belong to the lower tercile of the household asset index and to have younger household heads.

**Table 2:** Population characteristics of the study population by terciles of vegetable intake in the past month

Variables	Terciles of vegetable consumption in the past 30 days			Overall n = 187	*P*-trend
	Low (*n* = 62)	Moderate (*n* = 63)	High (*n* = 62)		
Vegetable intake (g/day)					
Mean ± SD	134.3 ± 42.9	285.5 ± 51.6	554.7 ± 143.3	324.6 ± 196.1	<.0001^*^
Median (IQR)	122.2 (92.3, 178.7)	289.0 (239.0, 325.8)	517.8 (428.2, 676.0)	289.0 (178.7, 428.2)	<.0001^*^
Frequency of intake^1^ (Mean ± SD)	7.0 ± 2.2	8.4 ± 2.0	9.4 ± 3.0	8.3 ± 2.6	<.0001
Age					
Mean ± SD	38.6 ± 11.6	38.1 ± 10.6	38.4 ± 11.9	38.1 ± 11.2	0.98
Median (IQR)	38.0 (29.0, 45.0)	36.0 (40.0, 45.0)	35.0 (30.0, 45.0)	36.0 (30.0, 45.0)	0.96
BMI					
Mean ± SD	21.9 ± 3.5	21.7 ± 3.4	23.0 ± 14.8	22.2 ± 9.0	0.69
Underweight	9.7	19.1	14.5	14.4	0.55
Normal weight	71.0	66.7	75.8	71.1	
Overweight	17.7	12.7	8.1	12.8	
Obese	1.6	1.6	1.6	1.6	
Household hunger					0.84
Little/no hunger	83.9	76.2	77.4	79.2	
Moderate hunger	12.9	19.1	19.4	17.1	
Severe hunger	3.2	4.8	3.2	3.7	
Vegetable variety score					
Mean ± SD	9.4 ± 3.0	10.6 ± 2.7	11.6 ± 3.3	10.5 ± 3.1	<.0003^*^
Low	45.1	17.5	19.4	27.2	<.0001^*^
Moderate	32.3	52.4	29.0	38.0	
High	22.6	30.2	51.6	34.8	
Household asset index					<.0001^*^
Low	17.7	44.4	59.7	40.6	
Moderate	46.8	34.9	35.5	39.0	
High	35.5	20.6	4.8	20.3	
Parity of woman	4.6 ± 2.4	4.7 ± 2.0	4.6 ± 2.2	4.6 ± 2.2	0.99
Dependency ratio	0.4 ± 0.2	0.4 ± 0.2	0.4 ± 0.2	0.4 ± 0.2	0.60
Marital status of woman					0.50
Currently Married	91.8	92.1	90.2	91.4	
Never married	3.3	0	4.9	2.7	
Formerly married	4.9	7.9	4.9	6.0	
Religious affiliation of woman					0.87
Islam	93.5	93.7	91.8	93.0	
Christianity	6.5	6.3	8.2	7.0	
Educational status of woman					0.37
Non-literate	91.9	85.7	83.9	87.2	
Literate	8.1	14.3	16.1	12.8	
Occupation of woman					0.74
Farmer	83.9	85.7	80.7	83.4	
Other	16.1	14.3	19.3	16.6	
Health status of woman					
Hypertension (yes)	4.8	3.2	8.1	5.4	0.43
Diabetes (yes)	1.6	0	1.6	1.1	0.55
Diabetes/hypertension	4.8	3.2	8.1	5.9	0.64
Drinking status	0	0	0	0	
Smoking status					0.33
Ever smoked	0	1.6	1.6	1.1	1.00
Sex of household head					0.80
Male	96.8	94.9	96.8	96.2	
Female	3.2	5.1	3.2	3.8	
Age of household head					
Mean ± SD	56.7 ± 13.3	51.1 ± 12.8	48.4 ± 12.1	52.0 ± 13.2	0.002^*^
Median (IQR)	56.0 (47.0, 62.0)	50.0 (40.0, 60.0)	48 (40.0, 56.0)	50.0 (40.0, 60.0)	0.004^*^
Educational status of household head					0.33
Non-literate	90.3	81.0	85.5	85.6	
literate	9.7	19.0	14.5	14.4	
Occupation of household head					0.50
Farmer	91.1	96.8	93.6	94.1	
Other	8.1	3.2	6.4	5.9	

Unless otherwise stated, values are frequencies and percentages; SD = standard deviation; ^1^Frequency of vegetable intake in the last month; *P-value is statistically significant at 5% level of significance

### The health-related quality of life (HR-QoL) and SF-36 subscales

The mean score and Alpha-Cronbach measure of reliability (α-Cronbach) for the HR-QoL were 69.5 ± 13.6 and 0.78 respectively. Also, the mean scores of the PH and MH domains were respectively 72.6 ± 17.4 and 66.4 ± 12.6 with α-Cronbach of 0.75 and 0.62 respectively. The mean score of the SF-36 subscales varied from 51.0 ± 10.4 for vitality/energy to 80.0 ± 17.4 for physical functioning. Lastly, the α-Cronbach for the measuring subscales varied from 0.08 for social functioning to 0.80 each for physical functioning and body pain respectively (Table 3).

**Table 3:** Mean scores and Alpha-Cronbach measure of the reliability of the health-related quality of life and its subscales among the women in fertile age

Scale	Mean ± SD	Alpha Cronbach (α)
HR-QoL	69.5 ± 13.6	0.78
Physical health component (PH)	72.6 ± 17.4	0.75
Mental health component (MH)	66.4 ± 12.6	0.62
Subscale of quality of life		
Physical functioning	80.0 ± 17.4	0.80
Role functioning/physical	74.0 ± 30.5	0.64
Body Pain	70.4 ± 26.1	0.80
General health	66.2 ± 17.9	0.74
Vitality /Energy	51.0 ± 10.4	0.69
Social functioning	75.4 ± 16.6	0.08
Emotional well-being	63.9 ± 14.2	0.66
Role functioning/emotional	75.5 ± 31.2	0.54

*SD* standard deviation, *HR-QoL* Health-related quality of life

#### The cross-sectional association between vegetable intake in the past month and the HR-QoL, as well its PH and MH components

In the crude model (Table 4), participants in the high tercile of vegetable intake had a significantly higher mean score for the HR-QoL compared to those in the lower tercile group (*P*-trend = 0.01). In the full multivariate model, the adjusted mean scores (95.5%) of the HR-QoL for participants in the moderate and high tercile groups of vegetable intake were significantly higher than those in the low tercile group (*P*-trend = 0.0003). Similarly, a significant increasing trend was observed in the adjusted mean scores of the PH domain for participants in the moderate and high tercile groups of vegetable intake compared to those in the low tercile group in the full multivariate model (*P*-trend = 0.002). Furthermore, in both the crude and full multivariate models, participants in the high tercile group of vegetable intake consistently obtained significantly higher MH scores (*P*-trend = 0.001) compared to those in the low tercile group of vegetable intake.

**Table 4:** The association between vegetable intake in the past month and health-related quality of life (HR-QoL) as well as its sub-scales among the women in fertile age

Model	Mean score (95% C.I) by terciles of vegetable intake			*P*-trend^1^
	Low (*n* = 62)	Moderate (*n* = 63)	High (*n* = 62)	
HR-QoL				
Crude model	65.9 (62.6, 69.3)	70.5 (67.2, 73.8)	72.1 (68.8, 75.4)^a^	0.01^*^
Multivariate model^2^	58.2 (52.0, 64.4)	64.3 (58.2, 70.4)^a^	67.4(61.3, 73.6)^a^	0.0003^*^
Physical health				
Crude model	68.3 (63.9, 72.6)	74.4 (70.1, 78.7)	75.4 (71.1, 79.7)	0.03^*^
Multivariate model^2^	57.8 (49.5, 66.1)	65.8 (57.6, 73.9)^a^	68.5 (60.3, 76.6)^a^	0.002^*^
Mental health				
Crude model	63.6 (60.5, 66.7)	66.6 (63.5, 69.6)	68.8 (65.7, 71.9)^a^	0.02^*^
Multivariate model^2^	58.5 (52.4, 64.6)	62.9 (56.8, 68.9)	66.4 (60.4, 72.5)^a^	0.001^*^
Subscales of the SF-36				
Physical functioning				
Crude model	75.9 (71.6, 80.2)	81.5 (77.3, 85.8)	82.3 (78.0, 86.6)	0.05^*^
Multivariate model^2^	61.7 (53.0, 70.4)	68.8 (60.2, 77.3)	71.4 (62.8, 80.0)^a^	0.01^*^
Role-physical				
Crude model	60.6 (53.4, 67.8)	76.9 (69.8, 84.1)^a^	85.1 (77.9, 92.3)^a^	<.0001^*^
Multivariate model^2^	49.5(34.7, 64.3)	69.8 (55.2, 84.3)^a^	80.0 (65.4, 94.6) ^a^	<.0001^*^
Bodily pain				
Crude model	70.5 (63.9, 77.1)	71.0 (64.5, 77.5)	69.6 (63.0, 76.2)	0.82
Multivariate model^2^	59.6 (46.1, 73.0)	60.2 (47.0, 73.5)	60.3 (47.1, 73.6)	0.90
General health				
Crude model	66.0 (61.4, 70.5)	68.2 (63.7, 72.7)	64.5 (60.0, 69.0)	0.59
Multivariate model^2^	60.5 (51.1, 69.9)	64.3 (55.0, 73.6)	62.1 (52.8, 71.4)	0.79
Vitality/energy				
Crude model	50.8 (48.2, 53.4)	50.2 (47.6, 52.7)	51.6 (49.0, 54.2)	0.62
Multivariate model^2^	49.9 (44.1, 55.6)	50.4 (44.7, 56.1)	52.5 (46.8, 58.2)	0.19
Social functioning				
Crude model	75.4 (71.1, 79.6)	75.6 (71.5, 79.8)	73.9 (69.7, 78.1)	0.61
Multivariate model^2^	75.3 (66.2, 84.3)	76.4 (67.5, 85.4)	73.4 (65.2, 83.1)	0.74
Role emotional				
Crude model	63.6 (56.1, 71.0)	76.2 (68.8, 83.5)^a^	87.1 (79.7, 94.5)^a^	<.0001^*^
Multivariate model^2^	48.0 (33.3, 62.7)	64.9 (50.4, 79.4)^a^	79.5 (65.0, 94.0)^a^	<.0001^*^
Emotional well-being				
Crude model	64.8 (61.2, 68.3)	64.3 (60.8, 67.9)	62.6 (59.1, 66.2)	0.39
Multivariate model^2^	60.9 (53.4, 68.3)	59.7 (52.4, 67.0)	59.6 (52.2, 66.9)	0.70

*HR-QoL* Health-related quality of life; *C.I* confidence interval; ^1^Test for trend based on median values in each tercile-ordinal variable; ^2^multivariate model was adjusted for age of woman, body-mass-index (BMI), parity, educational status of participant (literate/non-literate), occupation of participant (farmer/other), marital status of participant (never married, married and living with spouse and divorced/widow), age of household head, household hunger scale category and household asset index tercile; ^a^Statistically significantly higher (*P* < 0.05) than the low tercile category (Tukey-Kramer adjustment); *P-value is statistically significant at 5% level of significance

In the multivariate model, the occupation of the woman and household food security had reducing effects on the HR-QoL and its sub-scales; this effect was consistent for the PH, MH and sub-scales of the HR-QoL (Additional file 1: Table S1). The effects of the other possible confounding factors including age of the woman, BMI, parity, marital status, educational status of the woman, household asset index as well as the age of the household age were not statistically significant in the multivariate models (Additional file 1: Table S1).

### The cross-sectional association between vegetable intake in the past month and the SF-36 subscales

The crude and multivariate models in Table 4 also indicate that participants in the uttermost tercile of vegetable intake obtained significantly higher physical functioning scores compared to those in the low tercile (*P*-trend for multivariate model = 0.01). Likewise, participants in the moderate and high tercile groups of vegetable intake obtained significantly higher role-physical scores compared to those in the low tercile group (*P*-trend <.0001). Also, participants in the moderate and high tercile groups of intake had significantly higher scores for role-emotional compared to those in the low tercile group (*P*-trend <.0001).

### The association between vegetable variety score (VVS) and the HR-QoL and its domains

The adjusted mean score of the HR-QoL for participants in the uttermost VVS tercile was significantly higher than those in the moderate but not the low VVS tercile group (*P*-trend = 0.04) (Table 5).

**Table 5:** The association between vegetable variety score (VVS) and health-related quality of life (HR-QoL) as well as its sub-scales among the women in fertile age

Model	Mean score (95% C.I) by terciles of vegetable intake			*P*-trend^1^
	Low (*n* = 51)	Moderate (*n* = 71)	High (*n* = 65)	
HR-QoL				
Crude model	70.6 (66.9, 74.4)	67.1 (64.0, 70.3)	71.3 (68.0, 74.5)	0.59
Multivariate model^2^	62.1 (55.9, 68.3)	58.9 (52.6, 65.2)	66.3 (60.3, 72.3)^b^	0.04^*^
Physical health				
Crude model	76.1 (71.3, 80.9)	70.3 (66.3, 74.4)	72.6 (68.4, 76.9)	0.42
Multivariate model^2^	64.6 (56.2, 72.9)	59.3 (50.8, 67.8)	65.8 (57.7, 73.8)	0.56
Mental health				
Crude model	65.2 (61.8, 68.6)	63.9 (61.1, 66.8)	69.9 (66.9, 72.9)^b^	0.02^*^
Multivariate model^2^	59.6 (53.7, 65.6)	58.5 (52.5, 64.6)	66.8 (61.1, 72.6)^a, b^	0.001^*^
Subscales of the SF-36				
Physical functioning				
Crude model	79.4 (74.6, 84.1)	82.8 (78.7, 86.8)	77.3 (73.1, 81.5)	0.36
Multivariate model^2^	66.8 (58.0, 75.8)	69.1 (60.2, 78.0)	67.4 (58.9, 75.9)	0.93
Role-physical				
Crude model	76.0 (67.7, 84.2)	66.6 (59.7, 73.6)	81.1 (73.8, 88.4)^b^	0.18
Multivariate model^2^	62.8 (47.5, 78.1)	55.1 (39.5, 70.6)	74.7 (59.9, 89.5)^b^	0.02^*^
Bodily pain				
Crude model	77.4 (70.2, 84.5)	67.0 (61.0, 73.1)	68.5 (62.2, 74.9)	0.12
Multivariate model^2^	63.3 (50.2, 76.4)	53.1 (39.8, 66.4)	60.5 (47.8, 73.2)	0.72
General health				
Crude model	71.7 (66.8, 76.6)^c^	64.8 (60.7, 69.0)	63.5 (59.2, 67.9)	0.02^*^
Multivariate model^2^	65.4 (56.1, 74.7)	60.0 (50.6, 69.5)	60.7 (51.7, 69.7)	0.18
Vitality/energy				
Crude model	47.3 (44.5, 50.0)	49.6 (47.3, 51.9)	55.1 (52.7, 57.5)^a,b^	<.0001^*^
Multivariate model^2^	46.5 (41.0, 52.0)	49.7 (44.1, 55.2)	55.0 (49.7, 60.3)^a,b^	<.0001 ^*^
Social functioning				
Crude model	75.3 (70.7, 79.9)	73.6 (69.7, 77.5)	76.2 (72.1, 80.4)	0.66
Multivariate model^2^	74.0 (65.1, 83.0)	73.1 (64.0, 82.2)	77.0 (68.3, 85.7)	0.34
Role emotional				
Crude model	76.6 (68.1, 85.1)	70.0 (62.8, 77.2)	81.0 (73.5, 88.5)	0.28
Multivariate model^2^	61.4 (45.3, 77.5)	53.8 (37.5, 70.0)	71.6 (56.3, 86.9)^b^	0.05^*^
Emotional well-being				
Crude model	61.6 (57.7, 65.5)	62.6 (59.3, 65.9)	67.2 (63.7, 70.6)	0.03^*^
Multivariate model^2^	56.3 (49.1, 63.5)	57.5 (50.2, 64.8)	64.0 (57.1, 70.9)^a, b^	0.002^*^

*HR-QoL* Health-related quality of life, *C.I* confidence interval; ^1^Test for trend based on median values in each tercile-ordinal variable; ^2^multivariate model was adjusted for age of woman, body-mass-index (BMI), parity, educational status of participant (literate/non-literate), occupation of participant (farmer/other), marital status of participant (never married, currently married and formerly married), age of household head, household hunger scale category, household asset index tercile; ^a^Statistically significantly higher (*P* < 0.05) than the low tercile category (Tukey-Kramer adjustment); ^b^ Statistically significantly higher (*P* < 0.05) than the moderate tercile category (Tukey-Kramer adjustment); ^c^Statistically significantly higher (*P* < 0.05) than the high tercile category (Tukey-Kramer adjustment); *P-value is statistically significant at 5% level of significance

The PH domain was not associated with VVS in both the crude and multivariate models. On the contrary, a significant trend (*P*-trend = 0.001) was observed between the MH domain and the VVS terciles with participants in the high VVS tercile obtaining significantly higher scores compared to those in the low and moderate terciles. The multivariate models of the results in Table 6 also show a significant increasing trend between terciles of the VVS and role-physical (*P*-trend = 0.02), role emotional (*P*-trend = 0.05), emotional well-being (*P*-trend = 0.002) as well as vitality/energy (*P*-trend <.0001) domains of the SF-36.

Lastly, a decreasing trend was observed between the VVS terciles and the general health domain of the SF-36 in the crude model (*P*-trend = 0.02); but the observed trend was not statistically significant in the multivariate model (*P*-trend = 0.18).

## Discussion

### Association between vegetable intake, vegetable variety and the HR-QoL and its sub-domains

In agreement with Ruel et al. [2], the mean vegetable intake (324.6 ± 196.1 g/day) of the women in the present study was below the recommended level (at least 400 g/day) for the prevention of non-communicable diseases [1]. However, the mean vegetable intake was higher compared to the findings of Ruel et al. (137.3 g/day) [2]; this may be related to improved access as the study communities had irrigation facilities with year-round access to fresh vegetables. Furthermore, the communities had earlier been sensitised on the need to consume more of the fresh vegetables they cultivated in a nutrition education programme.

Overall, the results of the present study suggest a potential beneficial role of vegetable consumption on HR-QoL, PH and MH as well as the physical functioning, role-physical and role-emotional domains of the SF-36. Furthermore, the findings of the present study also suggest a beneficial role of the consumption of more varied vegetables on HR-QoL as well as MH and the emotional well-being, role physical, role emotional and vitality domains of the SF-36. Our findings may be emphasising the importance of improving the amount and variety of vegetable intake in future community and population-based programs or policies for improved mental and physical HR-QoL. Neither vegetable intake in the past month nor vegetable variety were associated with general health, social functioning and body pain in this study after adjusting for all potential confounders, suggesting that other factors rather than vegetable intake or vegetable variety may be reliable predictors of these scales.

Similar to other studies [35, 36], the HHS had a strong reducing effect on the HR-QoL and its sub-scales; this explains the large reductions in the scores of the HR-QoL and its sub-scales in the multivariate model. Women in households with severe hunger (mean difference = − 20.80, *P* < 0.0001), as well as those in households with moderate hunger (mean difference = − 7.35, *P* = 0.003), had significantly lower scores for the HR-QoL compared to their colleagues in households with little or no hunger (Additional file 1: Table S1); this effect was consistent for the PH, MH and most subscales of the SF-36. The HHS as used in the present study is an experience-based food insecurity scale [27]. Food insecurity may affect HR-QoL through different pathways, including chronic stress and anxiety [37] and nutritional status [11, 12, 38]. A lower household asset index also had a reducing but non-significant effect on the HR-QoL and its subscales. A lower household asset index implies a poorer socio-economic status which has been shown to be associated with a reduced quality of life in other studies [12, 39].

A direct comparison of our findings with other studies is difficult because of the differences in study design; most of the existing studies examined the combined effect of fruit and vegetable consumption on the HR-QoL with mixed results [11–13]. Our study is apparently the first to focus on the independent effect of vegetable consumption as well vegetable variety on the self-reported HR-QoL and its subscales in a developing context like Ghana. Nonetheless, among Chinese breast cancer survivors, Gong et al. [14] found that subjects consuming at least 250 g/day of vegetables had significantly higher scores for HR-QoL (adjusted mean difference = 4, *P* < 0.0001) as well as many of its sub-scales when compared to those with vegetable consumption lower than 250 g/day. Contrary to our findings, Matthews et al. [15] found vegetable intake to be associated with only the general health subscale of the SF-36 among older Australian women; in this study, vegetable variety was associated with general health perception (*P* = 0.03), vitality (*P* = 0.04) and social functioning (*P* = 0.04) for adult men but not women.

In the present study, we focused on the independent effect of vegetable intake because the consumption of fruits in Northern Ghana is generally poor [5]. In a study by Florkowski et al. [6], the consumption of home-grown fruits in the Northern Region of Ghana was only 0.1%, and income was found to be the most influential predictor of fruit consumption in this study. Most of the fruits in the region are imported from southern Ghana [4], thereby, increasing the cost with the consequence of poor access for deprived households particularly from rural communities. Considering that our study population was rural with a general low socio-economic status reflected in their poor educational and occupational status, we assumed fruit consumption may be scarce and may also not differ between the women in fertile age. For instance, a recent study that examined the effect of seasonality on the dietary diversity of school-aged children in the study area found that the consumption of vitamin C-rich fruits was less than 2% in both the dry and raining seasons; in this study, the consumption of vitamin A-rich fruits was significantly higher in the rainy season compared to the dry season (64% vs 0.9%, *P* = < 0.001), but was in part attributed the consumption of wild shea fruits (*Butyrospermum Parkii*) and mangoes, available only in the early part of the rainy season [40]. Overall, considering the lower fruit consumption and the seasonality association with it, vegetable intake perhaps remained the most critical exposure for the sampled population. Although our approach fails to consider the overall contribution of vegetable A and C-rich foods including vegetables and fruits, it seeks to contribute to the growing knowledge on the independent effect of vegetables on health and well-being.

It is worth noting that high consumption of vegetables is characteristic of the Mediterranean diet and although speculative, it is reasonable to assume that a higher vegetable intake may imply a higher adherence to the Mediterranean diet which has been associated extensively with HR-QoL [41–43]. Vegetables supply relatively high amounts of vitamins and minerals to the diet and are sources of phytochemicals that function as antioxidants, phytoestrogens, and anti-inflammatory agents with beneficial effects on general health and well-being [44–46]. Additionally, evidence from several systematic reviews [47–49] indicates that a dietary pattern rich in vegetables ensures micronutrient adequacy which improves health. In a Meta-analysis, Liu et al. [50] found that vegetable intake was independently associated with a lower risk of depression. Loef and Malachi [51] in a systematic review of cohort studies also concluded that increased intake of vegetables is independently associated with a lower risk of dementia in older age.

Although the biological mechanism for the beneficial effects of vegetable intake on the HR-QoL and its subscales is largely unknown, it may be due to the higher amounts of dietary fibre, vitamins, antioxidants (such as vitamin C and E) and polyphenols found in vegetables [46, 52, 53]. Antioxidants decrease the level of oxidative stress and thereby reduce the amount of DNA damage, neuronal cell death, and the aggregation of β-amyloid within the brain [54–56]; this is hypothesised to have consequences particularly for cognitive functioning and mental health. Furthermore, vitamin A is known to have an anti-inflammatory function [57, 58]; although this mechanism is also not well understood, subjects with a better vitamin A status may have a lower risk of inflammation and consequently a better health status.

### Strengths and limitations of the study

Several strengths and limitations should be taken into account when interpreting the findings of the present study. First and foremost, the current study is ostensibly the first of its kind to study the association between vegetable intake and the self-reported HR-QoL in the Ghanaian population. Additionally, it is seemingly the first to give an account of the association between a score of vegetable variety and the HR-QoL in Ghana.

The SF-36 is self-reported and may be susceptible to reporting bias such as social desirable answers including under or over-reporting; however the SF-36 is a thoroughly validated and widely used scale for self-reported HR-QoL [22–24], which could explain that the Cronbach measure of reliability for the HR-QoL (α = 0.78) was adequate according to the standard criteria [29]. Similarly, except the social functioning domain (α = 0.08), the Cronbach-α of the SF-36 domains were acceptable [29].

In our present study, the use of a pre-tested FFQ and training of participants helped ensure that consumption was recorded more appropriately. However, recall bias and measurement errors are associated with FFQs, but we tried to minimise any recall bias through the use of household measures, food models and picture aids. It should be noted that the FFQ has strengths in its ability to minimise the day-to-day variation in dietary intake by assessing usual intake; most importantly, the FFQ has strengths in discriminating individual dietary patterns and ranking them accordingly. Hence the FFQ was a suitable method for the assessment of usual vegetable intake in the current study.

Residual confounding in observational studies is always possible despite extensive statistical adjustments [31]; hence, though we adjusted for several potential confounders, we cannot rule out the effect of residual confounding. Notably, physical activity is often adjusted for in many related studies [16, 42, 43], but the present study did not include an assessment of physical activity. Nonetheless, considering that the women were generally farmers engaged in labour-intensive agriculture, their physical activity was most likely high on the average.

The main limitation of the current study is its cross-sectional design; inference of a possible causality is speculative since it is not possible to determine whether adherence to a higher vegetable intake or VVS preceded a better HR-QoL or that those with a better HR-QoL simply consumed more vegetables or more varieties of vegetables. A prospective design would better be equipped to address this problem. We, therefore, limit the interpretation of our findings to describing the observed associations.

At last, our results are based on a cohort of rural women in fertile age from the Northern Region of Ghana; caution is therefore needed in extending the results presented here to larger contexts since our sample could be considered to be more representative of rural women in fertile age in the Northern Region of Ghana.

## Conclusion

The present study contributes to the scarce literature on the association between vegetable intake and self-reported HR-QoL. The findings suggest a significant positive association between higher vegetable intake and self-reported HR-QoL including the physical and mental health components and 3 of the SF-36 domains (physical functioning, role-physical and role-emotional). Our data further suggest a significant positive association between a high vegetable variety and self-reported HR-QoL, mental health as well as the emotional well-being and vitality domains of the SF-36. The present study is essential in further examining the hypothesis on the cause-effect association between vegetable consumption and HR-QoL and in planning intervention strategies that may be helpful in preventing mental and physical health decline. Our findings emphasise the importance of improving vegetable intake in future community and population-based programs or policies to improve quality of life towards healthy ageing.

## Additional file

Additional file 1: **Table S1.** Multivariate general linear regression (GLM) of the association between vegetable consumption and HR-QoL, MH and PH among women in fertile age (DOCX 15 kb)

## Abbreviations

BMI: Body mass index
FFQ: Food frequency questionnaire
HHS: Household hunger scale
HR-QoL: Health-related quality of life
KMO: Kaiser-Meyer-Olkin
MH: Mental health
PH: Physical health
SF-36: Short form health survey 36
VVS: Vegetable variety score
