# Supplementary material for: Higher vegetable intake and vegetable variety is associated with a better self-reported health-related quality of life (HR-QoL) in a cross-sectional survey of rural northern Ghanaian women in fertile age
Source: BMC Public Health. 2018 Jul 27;18:920. doi: 10.1186/s12889-018-5845-3 (PMC6062975; doi:10.1186/s12889-018-5845-3)
Supplement: Supplementary file 1 — Table S1. Multivariate general linear regression (GLM) of the association between vegetable consumption and HR-QoL, MH and PH among women in fertile age (DOCX 15 kb) [file 12889_2018_5845_MOESM1_ESM.docx]

**Table S1: Multivariate general linear regression (GLM) of the association between vegetable consumption and HR-QoL, MH and PH among women in fertile age**

| **Variable** | **HR-QoL Score** | | | **MH Score** | | | **PH Score** | | |
| --- | --- | --- | --- | --- | --- | --- | --- | --- | --- |
|  | **Estimate** | **S.E (estimate)** | **P-value** | **Estimate** | **S.E (estimate)** | **P-value** | **Estimate** | **S.E (estimate)** | **P-value** |
| Tercile of Vegetable intake |  |  |  |  |  |  |  |  |  |
| Low | Ref (0) |  |  | Ref (0) |  |  | Ref (0) |  |  |
| Moderate | 6.12 | 2.23 | 0.007^*^ | 7.93 | 2.41 | 0.001^*^ | 7.95 | 2.97 | 0.008^*^ |
| High | 9.28 | 2.45 | 0.0002^*^ | 4.36 | 2.20 | 0.049^*^ | 10.64 | 3.26 | 0.001^*^ |
| Age of women (years) | -0.18 | 0.12 | 0.12 | -0.15 | 0.12 | 0.20 | -0.22 | 0.16 | 0.17 |
| Parity | -0.46 | 0.48 | 0.34 | -0.42 | 0.47 | 0.38 | -0.50 | 0.64 | 0.43 |
| BMI of woman | 0.02 | 0.10 | 0.86 | 0.07 | 0.10 | 0.51 | -0.03 | 0.14 | 0.83 |
| Occupation of woman |  |  |  |  |  |  |  |  |  |
| Farmer | Ref (0) |  |  | Ref (0) |  |  | Ref (0) |  |  |
| Other | -7.80 | 2.45 | 0.002^*^ | -5.24 | 2.42 | 0.03^*^ | -10.35 | 3.27 | 0.002^*^ |
| Educational status of woman |  |  |  |  |  |  |  |  |  |
| Literate | 2.59 | 2.95 | 0.38 | 0.66 | 2.91 | 0.82 | 4.51 | 3.92 | 0.25 |
| Non-literate | Ref (0) |  |  | Ref (0) |  |  | Ref (0) |  |  |
| Marital status of woman |  |  |  |  |  |  |  |  |  |
| Never married | 0.42 | 0.72 | 0.95 | -2.66 | 7.10 | 0.71 | 3.50 | 9.60 | 0.72 |
| Married | -3.10 | 4.36 | 0.48 | -5.16 | 4.30 | 0.23 | -1.05 | 5.81 | 0.86 |
| Formerly married | Ref (0) |  |  | Ref (0) |  |  | Ref (0) |  |  |
| Household hunger scale |  |  |  |  |  |  |  |  |  |
| Little/ no hunger | Ref (0) |  |  | Ref (0) |  |  | Ref (0) |  |  |
| Moderate hunger | -7.35 | 2.44 | 0.003^*^ | -4.93 | 2.40 | 0.042^*^ | -9.76 | 3.25 | 0.003^*^ |
| Severe hunger | -20.80 | 4.45 | <0.0001^*^ | -15.76 | 4.39 | 0.0004^*^ | -25.83 | 5.93 | <0.0001^*^ |
| Household asset index |  |  |  |  |  |  |  |  |  |
| Low | -2.15 | 2.67 | 0.43 | -3.77 | 2.63 | 0.15 | -0.53 | 3.56 | 0.88 |
| Moderate | -1.24 | 2.46 | 0.62 | -3.75 | 2.42 | 0.12 | 1.27 | 3.28 | 0.70 |
| High | Ref (0) |  |  | Ref (0) |  |  | Ref (0) |  |  |
| Age of household age (years) | 0.13 | 0.08 | 0.11 | 0.10 | 0.08 | 0.21 | 0.17 | 0.11 | 0.13 |

HR-QoL, health-related quality of life; MH, mental health sub-scale of the heath-related quality of life; PH, physical health sub-scale of the health related quality of life. ^*^Statistically significant at 5% alpha
